# Water Channels Aquaporin 4 and -1 Expression in Subependymoma Depends on the Localization of the Tumors

**DOI:** 10.1371/journal.pone.0131367

**Published:** 2015-06-26

**Authors:** Susan Noell, Petra Fallier-Becker, Andreas F. Mack, Maike Hoffmeister, Rudi Beschorner, Rainer Ritz

**Affiliations:** 1 Department of Neurosurgery, University of Tuebingen, Tuebingen, Germany; 2 Institute of Pathology and Neuropathology, University of Tuebingen, Tuebingen, Germany; 3 Institute of Clinical Anatomy and Cell Analysis, University of Tuebingen, Tuebingen, Germany; 4 Department of Neurosurgery, University of Marburg, Marburg, Germany; Hungarian Academy of Sciences, HUNGARY

## Abstract

**Background:**

We analyzed aquaporin 4 and -1 expression in subependymomas, benign and slow growing brain tumors WHO grade I. Ten subependymoma cases were investigated, five of the fossa inferior and five of the fossa superior.

**Methods and Results:**

Using immunohistochemistry, we observed different aquaporin expression patterns depending on localization: aquaporin 4 and -1 were detected in infratentorial subependymomas in the entire tumor tissue. In contrast, supratentorial subependymomas revealed aquaporin 4 and -1 expression only in border areas of the tumor. PCR analyses however showed no difference in aquaporin 4 expression between all subependymomas independent of localization but at higher levels than in normal brain. In contrast, aquaporin 1 RNA levels were found to be higher only in infratentorial samples compared to supratentorial and normal brain samples. The reason for the different distribution pattern of aquaporin 4 in subependymomas still remains unclear. On the cellular level, aquaporin 4 was redistributed on the surface of the tumor cells, and in freeze fracture replicas no orthogonal arrays of particles were found. This was similar to our previous findings in malignant glioblastomas. From these studies, we know that extracellular matrix molecules within the tumor like agrin and its receptor alpha-dystroglycan are involved in forming orthogonal arrays of particles. In subependymomas neither agrin nor alpha-dystroglycan were detected around blood vessels.

**Conclusions:**

Taken together, we show in this study that in the benign subependymomas aquaporins 1 and 4 are dramatically redistributed and upregulated. We speculate that extracellular environments of infra- and supratentorial subependymomas are different and lead to different distribution patterns of aquaporin 4 and -1.

## Introduction

Several glial-derived tumors are known to occur in the central nervous system. They range in malignancy from benign to highly malignant. Most of them are characterized by an altered expression of glial-specific proteins such as fibrillary acidic protein (GFAP), and other proteins like the water channel aquaporin 4 (AQP4). The redistribution of these water channels in highly malignant gliomas has been taken as an indicator of a disturbed blood-brain barrier. In malignant gliomas new therapeutic approaches e.g. monoclonal human IgG antibodies against extracellular aquaporin-4 domains exist by now[1].

For a better understanding of the mechanisms underlying the distribution of aquaporins (AQP) in comparison of extremely slow growing brain tumors like subependymoma (SE) to faster and more invasive growing tumors e.g. high grade ependymomas (WHO grade II-III) or malignant gliomas (WHO grade III-IV), we addressed in the present study the question whether an altered distribution of AQPs is also present in benign SEs. In these benign tumors, edema formation, invasive growth, and formation of recurrent tumors play no significant roles as in malignant tumors

Aquaporins (AQP) are water channels discovered by Peter Agre [2,3] and George Benga [4,5]. Of the at least 13 different water channels in mammals,the main water channel expressed in the healthy brain is AQP4 found in astrocytes and ependymal cells [6,7]. In astrocytes, AQP4 expression is mostly restricted to endfoot membranes contacting the basal lamina of capillaries, containing components which are important for the specific morphology of AQP4, the orthogonal arrays of particles (OAP) [8–11]. Astrocytic endfoot membranes are part of the glia limitans perivascularis et superficiales and show the typical formation of orthogonal arrays of particles (OAPs) [12]. Parenchymal astrocytic membranes without contact to the basal lamina however reveal only a small number of OAPs [13]. Under pathological conditions like glioblastoma, AQP4 is up regulated [14–16]. Here, the OAP-structure is lost or altered, so that in freeze fracture replicas the channel appears in single proteins [17] or in hypoxic protein clusters in the membranes [18]. Whether this is also the case in benign SEs is part of the present investigation.

Our previous studies have shown that the expression of AQP4 in glioma cells and its morphological appearance depend on the environmental milieu, especially on components of the extracellular matrix (ECM) of the brain region itself, or within the tumor [17,19]. One important component of the ECM is the heparan-proteoglycan agrin cleaved by the matrix-metallo-proteinase MMP3, in glioblastomas [20]. Agrin is connected to a membrane-spanning molecule complex, called dystrophin-dystroglycan complex (DDC [21]). Agrin binds to α-dystroglycan in the astrocytic membrane and is indirectly connected to AQP4 via ß-dystroglycan, dystrophin and α-syntrophin. If agrin or dystroglycan are knocked out, formation of OAPs is defective [10,22]. Agrin is a substrate of MMP3, dystroglycan of MMP2 and 9, possibly the reason for the absence of agrin and dystroglycan around vessels in glioblastomas [17].

**Fig 1:**
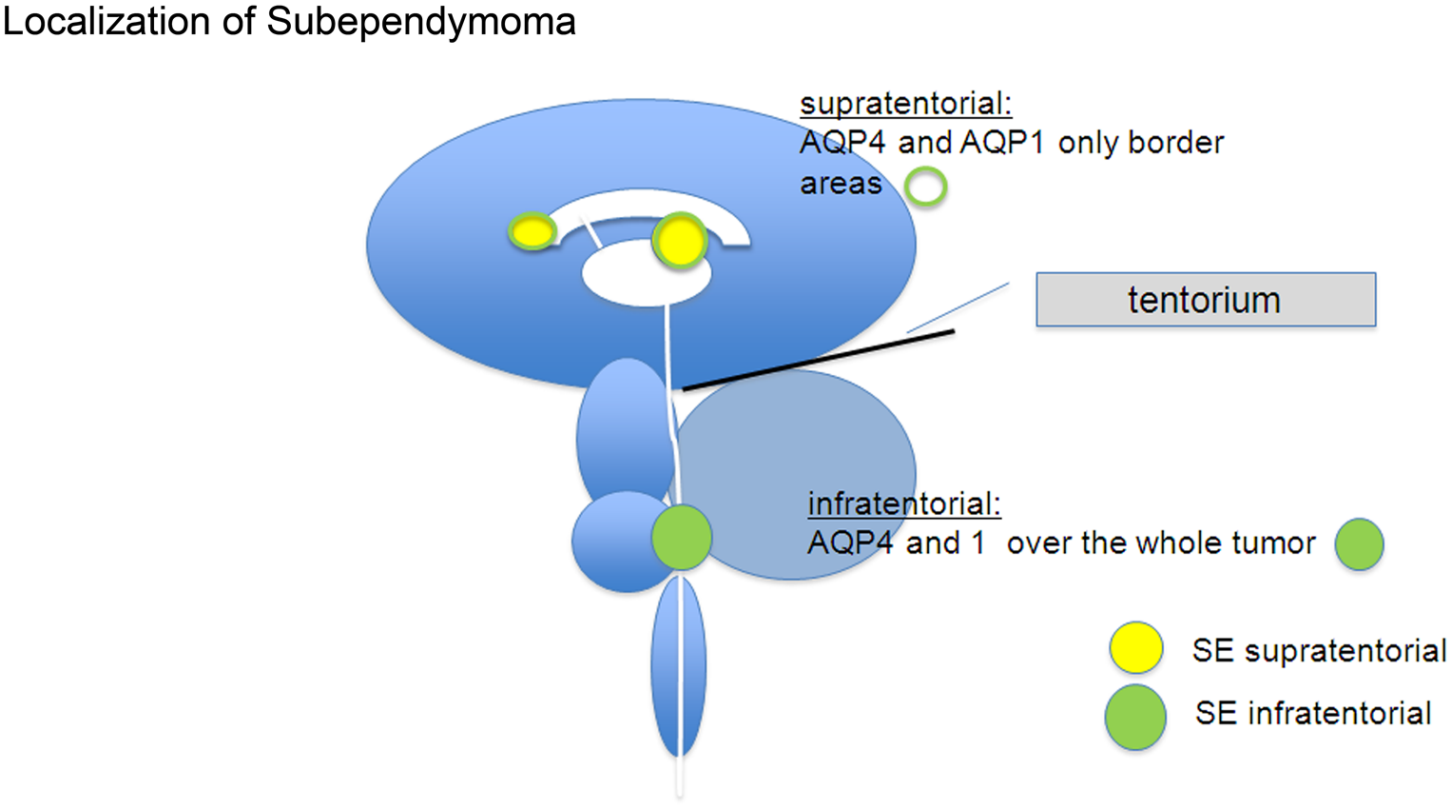
Highly schematic diagram indicating the location of subependymomys (SEs) in the human brain. The main findings on AQP distributions are also indicated.

Besides alteration in AQP4 expression, the water channel AQP1 is upregulated under pathological conditions, e.g. in glioma cells and reactive astrocytes as well as in reactive brain endothelial cells [23]. AQP1 is expressed in the epithelial cells of the choroid plexus but not in other healthy ependymal cells nor in healthy astrocytes[24]. AQP1 is capable of transporting oxygen as well as water across cell membranes which is an important function of being mostly expressed in red blood cells and in the choroid plexus In brain tumors like ependymomas AQP4 as well as AQP1 are found but AQP4 was only expressed in ependymomas when tumors grow in the fossa inferior of the brain, but not in supratentorial regions [25]. Ependymomas (E) belong to glial tumors and are graded in WHO grade II and III. They are more aggressive than SEs and tend to develop recurrent tumors. SEs are graded WHO grade I. They are benign and grow very slowly. Transformations from WHO grade I to WHO grade II are extremely rare [26]. Two thirds of these tumors are found infratentorial and grow from the bottom of the fourth ventricle. One third grows supratentorial and has mostly contact to the ventricle system (Fig 1).

At first we focused in this study on different expression patterns of AQP4 and -1 in SEs and the possible correlation to the localization of the tumor. We found AQP4 and AQP1 were expressed that in infratentorial SEs throughout the entire tumor whereas in supratentorial SEs AQP4 and -1 were detected only in peripheral regions of the tumor but not in the center. Secondly, the tissue within the tumors was analyzed using the freeze fracture technique. SEs did not reveal any OAPs. This is consistent with the finding that neither agrin nor α-dystroglycan were expressed in SEs around the vessels although the impairment of the blood-brain barrier is less than in glioblastoma

## Material and Methods

### Patients

The project and patients’ consent procedures were approved by the ethics committee of the medical faculty of the Eberhards-Karl University of Tuebingen. The ethics committee waived the need for consent. We investigated 5 tumor samples from subependymomas of the fourth ventricle and 5 SE tumor samples growing supratentorial with relation to the first to third ventricle (Fig 1). All patients were treated in the department of Neurosurgery Tübingen between 1999 and 2012 (Table 1). Control brain tissue that was removed in order to facilitate the approach for tumour resection was used from one patient suffering from a meningioma.

**Table 1:** Patient history.

patient number	sex	age at time of surgery (years)	tumor localization
1	m	71	infratentorial near IV. ventricle
2	f	42	infratentorial near IV. ventricle
3	f	42	infratentorial near IV. ventricle
4	m	75	infratentorial near IV. ventricle
5	m	43	infratentorial near IV. ventricle
6	m	50	supratentorial near right frontal ventricle
7	m	59	supratentorial near right frontal ventricle
8	f	44	supratentorial near both frontal ventricle
9	f	45	supratentorial near both frontal ventricle
10	m	55	supratentorial near left frontal ventricle

### Immunohistochemistry

Obtained tumor samples were dissected and formalin fixed overnight followed by paraffin embedding. 5 μm thick sections were deparaffinized and rehydrated. Nonspecific peroxidase was blocked with 3% hydrogen peroxide. Sections were subjected to a heat induced epitope retrieval in Citratebuffer (pH 6.0). Subsequently immunostaining was performed: Primary antibodies (listed in Table 2) were diluted in antibody diluent (Zytomed Systems, Berlin, Germany) and applied on sections over night at 4°C in a humid chamber. Immunostaining continued by using the Zytochem Plus HRP Polymer Kit (Zytomed Systems, Berlin, Germany) and peroxidase activity was visualized with DAB-substrate kit (Zytomed Systems, Berlin, Germany). Hematoxylin (Merck, Darmstadt, Germany) was chosen for counterstaning before dehydrating and coverslipping in Roti Histokitt (Carl Roth GmbH, Karlsruhe, Germany). Primary antibodies were omitted for negative control. Sections were analyzed and documented with a Mirax slide scanner (Zeiss, Germany) For immunfluorescence staining we used the secondary goat-anti-mouse, or goat-anti-rabbit antibodies labeled with cyanin-derivative dye Cy2, Cy3 (Dianova, Hamburg, Germany), or goat- anti-mouse Alexa-488 (Molecular Probes/Life Technologies, Darmstadt, Germany). For controls the primary antibody was omitted. Sections were analyzed with a confocal laser scanning microscope (LSM510 META with an Axioplan 2 microscope stand, Zeiss, Göttingen/Jena, Germany).

**Table 2:** Primary antibodies used in human subependymomas.

Primary antibodies	Cat.No.	Source
Agrin, rabbit, polyclonal	Ab 85174	Abcam, Cambridge, UK
AQP1, mouse, monoclonal	Sc-32737	Santa Cruz, Heidelberg, Germany
AQP4, rabbit, polyclonal	Sc-20812	Santa Cruz, Heidelberg, Germany
α–dystroglycan, rabbit polyclonal	Sc-28534	Santa Cruz, Heidelberg, Germany
GFAP, mouse, monoclonal	MAB 360	Millipore, Darmstadt, Germany
GFAP, rabbit, polyclonal	Z 0334	Dako, Hamburg, Germany
MMP-2, goat, polyclonal	AF 902	R&D, Wiesbaden, Germany
MMP-3, goat, polyclonal	AF 513	R&D, Wiesbaden, Germany
MMP-9, rabbit, polyclonal	ABD-8C0275	Biomol, Hamburg, Germany

### Electron microscopy and freeze-fracturing

For conventional electron microscopy, tissues were fixed with 2.5% glutaraldehyde (Paesel-Lorei, Frankfurt, Germany) buffered in 0.1 M cacodylate buffer (pH 7.4). Thereafter, the tissues were postfixed in the identical fixative for additional 4 hours, and then stored in cacodylate buffer until further processing as previously described in detail. Freeze-fracturing of the glutaraldehyde-fixed tissues was performed as described before [27]. Ultrathin sections and replicas were analyzed with a Zeiss EM 10 electron microscope (Zeiss, Oberkochen, Germany).

### RNA extraction

Formalin-fixed, paraffin-embedded (FFPE) tumor specimens of the above mentioned subependymomas were retrieved from the surgical pathology files of the Department of Neuropathology of the Institute of Pathology of Tuebingen

RNA of the samples was extracted manually with the chloroform/phenol extraction method. Shortly, FFPE sections were deparaffinized, followed by proteolysis through proteinase K overnight. The following day, RNA was extracted through chemical precipitation (chloroform/phenol), washed with 70% ethanol and dissolved in DNase/RNase free water. RNA quantification was performed with the NanoDrop ND-2000 spectrophotometer.

### cDNA-synthesis

1 μg RNA was used for reverse transcription with the High-Capacity cDNA RT Kit (Applied Biosystems) for mRNA analysis in a total volume of 20 μl. In detail, 1 μg RNA (dissolved in a total volume of 10 μl RNase-free water) was mixed with 3.2 μl RNase-free water, 2 μl 10x RT buffer, 2 μl 10x RT Random Primers, 0.8 μl 25x dNTP Mix (100 nM), 1 μl MultiScribe Reverse Transcriptase (50 U/μl) and 1 μl RNase Inhibitor (20 U/μl). cDNA synthesis was carried out as follows: 10 min at 25°C, 37°C for 2h and 5 sec at 85°C.

### Primer design

Intron-spanning oligonucleotides were designed with the program Primer3Plus (http://primer3plus.com/cgi-bin/dev/primer3plus.cgi). In Table 3 the primer sequences and the product sizes are shown. The specificity of the primers was checked with the program Primer-BLAST (http://www.ncbi.nlm.nih.gov/tools/primer-blast/).

**Table 3:** Primer sequences and product sizes.

Primer	Sequence	Product size
HPRT1 Ex6 for	tgacactggcaaaacaatgc	
HPRT1 Ex7 rev	ttcgtggggtccttttcacc	101bp
AQP1 Ex2 for	cgtgaccttggtggctcag	
AQP1 Ex3 rev	ggaccgagcagggttaatcc	105bp
AQP4 Ex4 for*	aacggactgatgtcactggc	
AQP4 Ex5 rev*	aaaggatcgggcgggattc	113bp

*The AQP4 Primers were designed to amplify both isoforms of AQP4 (M1+M23)

### PCR

PCR for the AQP4- and AQP1 products as well as HPRT1 was conducted with 2.5 μl 10x PCR Buffer I (15 mM MgCl_2_), 0.25 μl AmpliTaq Gold DNA Polymerase (both Life Technologies, Carlsbad, CA, USA) 1 μl Primer each, 0.5 μl dNTPs (10 mM each; Qiagen, Hilden, Germany), and 100 ng cDNA in a total volume of 25 μl. The reaction mixtures were initially heated at 95°C for 5 min to activate the polymerase, followed by 40 cycles including a denaturation step at 94°C for 45 sec, an annealing step at 50°C for 45 sec and an elongation step at 72°C for 45 sec, with a final elongation step at 72°C for 5 min. Negative control (H_2_O) and positive control (lung tissue for AQP4 and one Normal Brain sample for AQP1; Clontech Laboratories, CA, USA) were included.

The PCR products (each 5 μl) were separated by gel electrophoresis on a 1,5% agarose gel, stained with GelRed (Biotum, Hayward, CA, USA) and illustrated by UV irradiation.

### RT-PCR

Total RNA (1μg) was reversely transcribed using the High-Capacity cDNA RT Kit with RNA Inhibitor (Applied Biosystems). Real-time PCRs (10 μl Real-Time SYBR Green PCR master mix, 1 μl (20ng) diluted reverse transcription product, 2 μl each of Primer (Table 3) and 7 μl DNase/RNase free water) were carried out in triplicates in a LightCycler 480 II (Roche, Basel, Switzerland) under following conditions: 95°C for 15 min followed by 40 cycles of 94°C for 15 sec, 55°C for 30 sec and 70°C for 30 sec. Two normal brain controls were included in every run. A melting curve analysis was carried out to assess the specificity of the amplified PCR product.

### Data analysis

The raw Cp values were imported into Microsoft Excel (Microsoft Corp., Seattle, USA). The AQP1 and AQP4 expression was analyzed using the comparative ΔCp method, where ΔCp = (Cp _candidate target_ - Cp _reference RNA_). HPRT1 was used as endogenous reference gene for the analysis.[28]

### Statistical analysis

Statistical analyses of differential gene expression between infra- and supratentorial SE as well as between healthy brain samples and SE samples were performed using the program GraphPad Prism4. Two-tailed *T-Test* was applied to the different statistical comparisons.

## Results and Discussion

**Fig 2:**
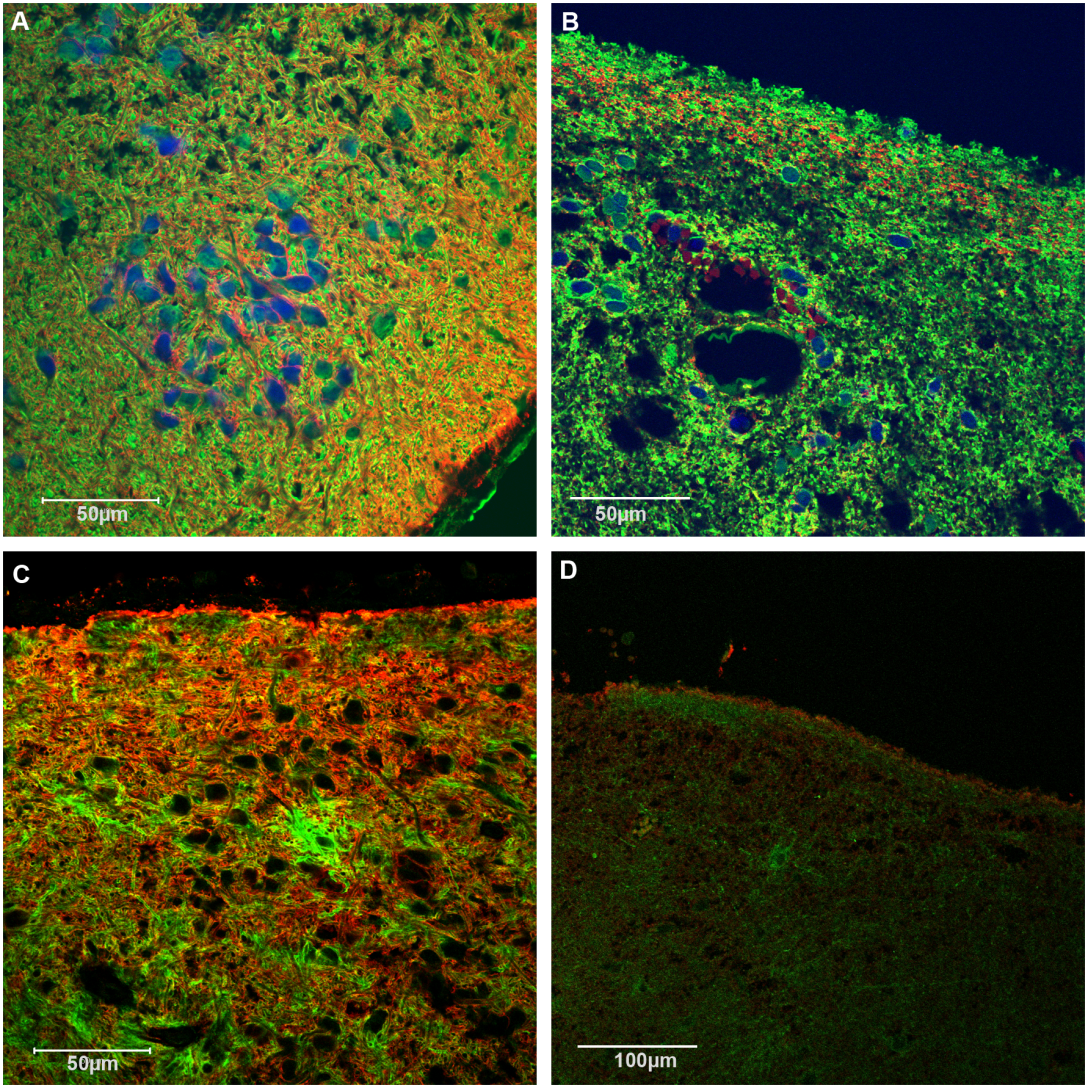
Immunofluorescent double staining for aquaporins in SEs. Double staining of GFAP- (green) and AQP4 (red) in infratentorial (A, patient 5) and supratentorial (B patient 6) SE. Dapi stained nuclei in blue. Double staining of AQP4 (red) and AQP1 (green) in infratentorial (C patient 4) and supratentorial (D patient 6) SE. AQP 1 and AQP4 positive cells are more or less evenly distributed in infratentorial tumors, in contrast they were found only in the border region of supratentorial tissue. Bar: 50μm (A, B, C); 100μm (D).

**Fig 3:**
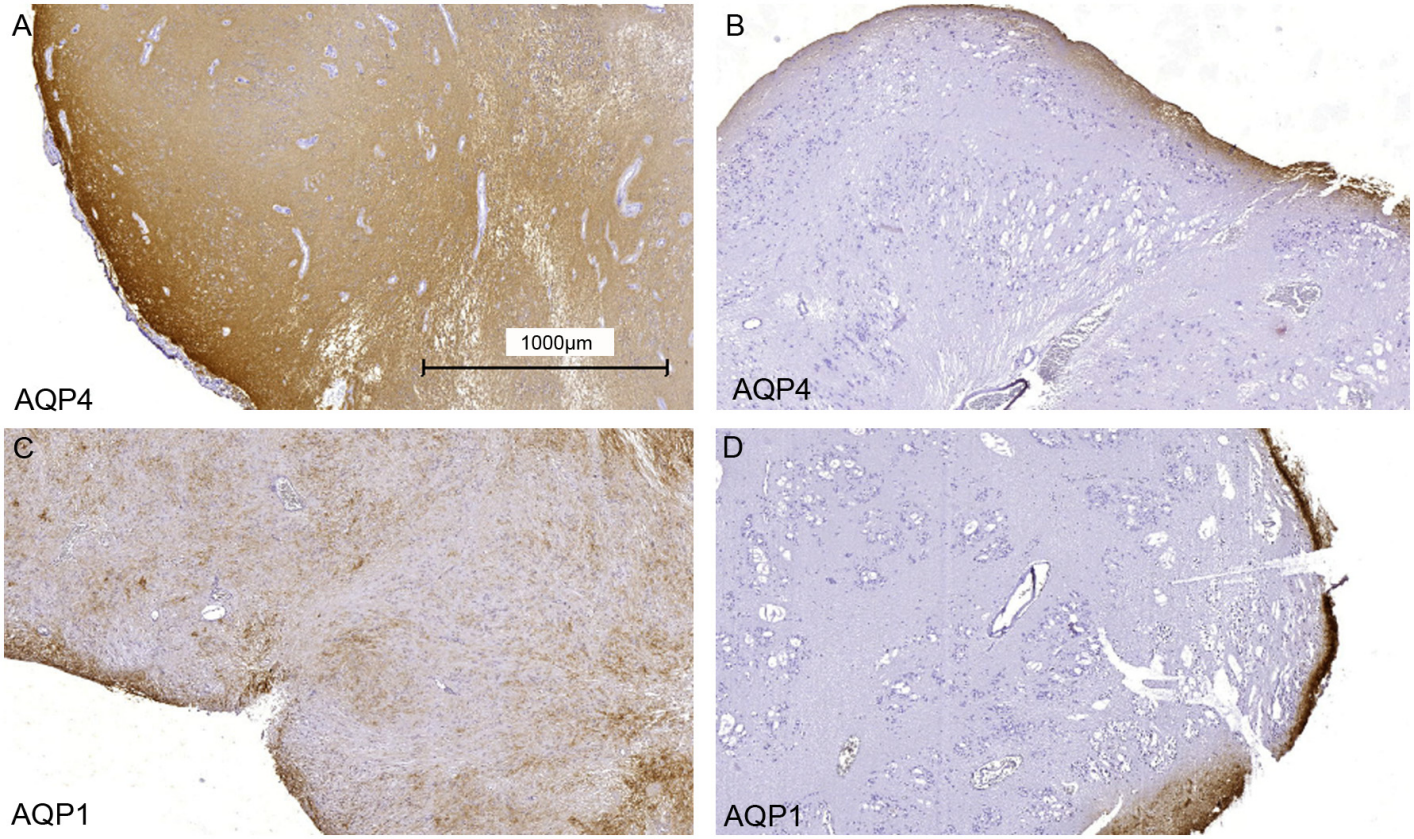
Overview immunhistological staining of AQP4- and -1 in SE. Infratentorial SE tissue shows AQP4 and -1 immunoreactivity over the whole tumor (A patient 5, C patient 4), whereas supratentorial SE shows AQP4 and -1 staining almost always at the natural border regions of the tumor (B patient 6, D patient 8). Bar 1000μm.

First we tested the expression of typical glial markers in SE tissues by immunochemistry. Like in other glial derived tumors GFAP expression was positive in all SEs. Double staining with antibodies against GFAP and AQP4 revealed evenly distributed immunofluorescence for GFAP, throughout all tumors (Fig 2A and 2B), whereas AQP4 immunofluorescence appeared evenly in infratentorial tumors only (Fig 2A). In supratentorial SEs, AQP4 staining was restricted to the peripheral region (Fig 2B). AQP4- and AQP1—immunostaining revealed a more or less even distribution in infratentorial SE tumors of the fourth ventricle of the fossa inferior as shown in Fig 2A, 2C, Fig 3A, 3C and S1 Fig. In contrast, in SEs growing supratentorial in other parts of the ventricle system AQP4 and -1 mostly appeared in the margin of some tissue samples in accordance with the tumor margin (Fig 2B and 2D, Fig 3B and 3D and S2 Fig).

**Fig 4:**
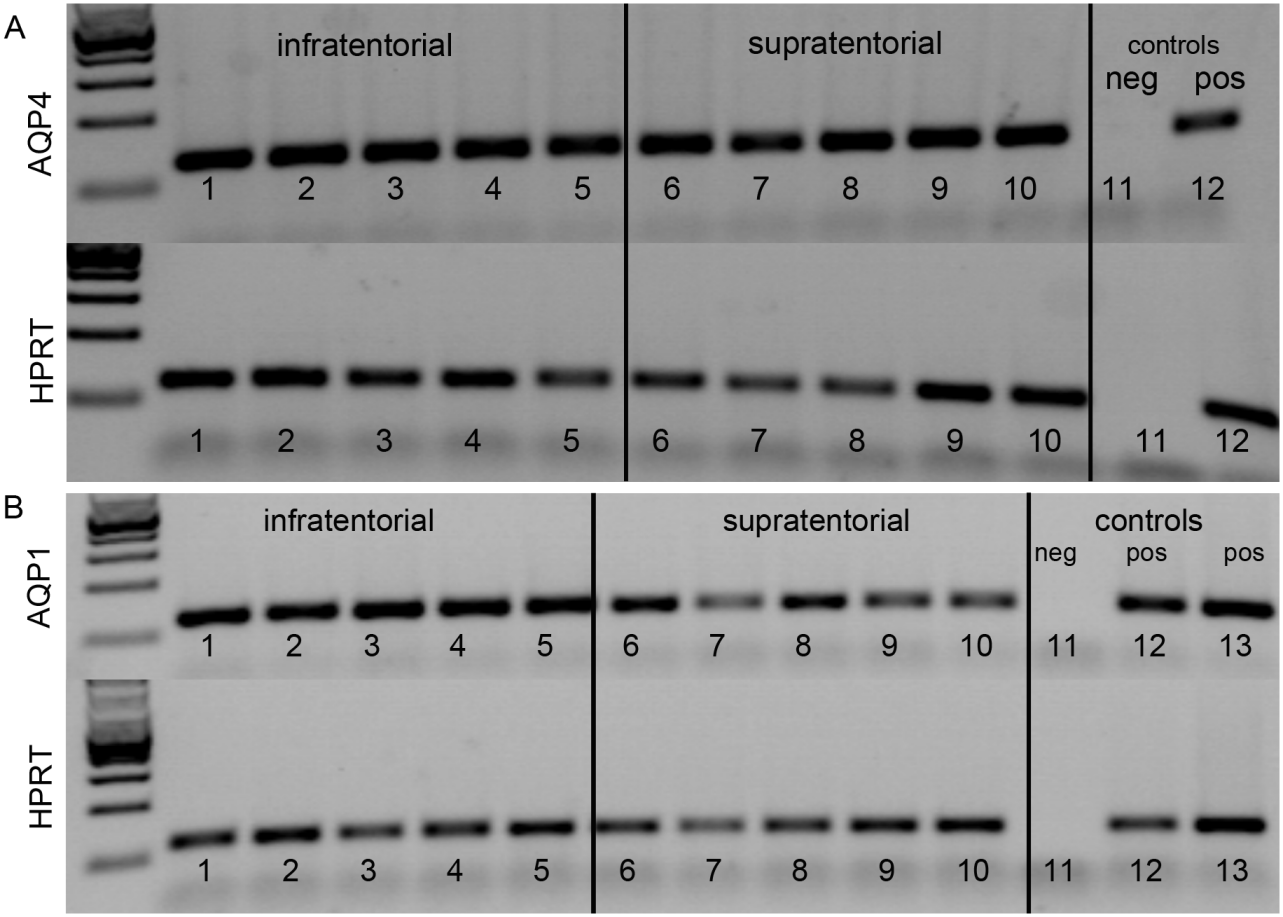
PCR analysis of AQP4 and AQP1 infratentorial (patients 1–5 or lane 1–5 respectively) and supratentorial (patients 6–10 or lane 6–10) respectively SE. A: In every location AQP4 (Exon 4–5) was expressed. Lane 11: negative control (H_2_O), lane 12 positive control (lung). HPRT lane 1–12. B: In every location AQP1 was expressed. The infratentorial SE samples showed very distinct bands in the gel, whereas the AQP1 expression in supratentorial SEs varied and where less distinct. Lane 11: negative control (H_2_O), lane 12 and 13 positive control (Normal Brain). HPRT1 lane 1–13.

**Fig 5:**
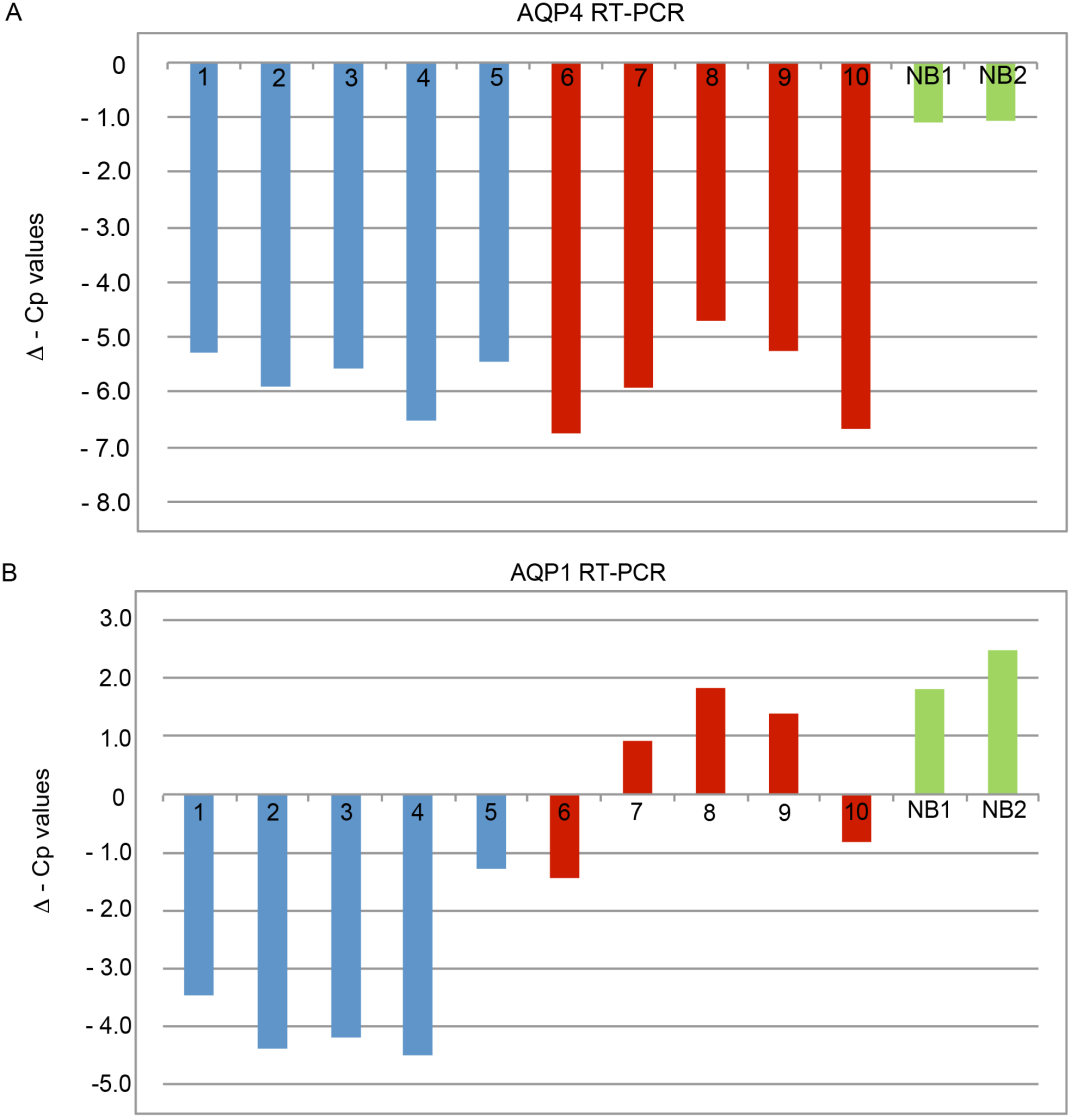
AQP4 and AQP1 expression (normalized to HPRT1) in SE and normal brain. A: AQP4 was upregulated in both SE subgroups (ΔCp -4.7 to -6.75) compared to normal brain (ΔCp -1.06/-1.09) (B). (ΔCp-values <-1 mean upregulation, >1 mean downregulation). B: Infratentorial SE show upregulated AQP1 expression (ΔCp -1.27 to -4.49) in contrast to supratentorial SE (ΔCp 1,83 to -1.44) and Normal Brain (ΔCp 1.81/2.48), which show a slight downregulation (A). Blue: patients 1–5 infratentorial SE, red: patients 6–10 supratentorial SE, green: Normal Brain as positive controls.

To investigate AQP4 expression on the RNA level, we performed PCR analysis with cDNAs of five infratentorial growing SEs (Fig 4A above lane 1–5) and five supratentorial SEs (Fig 4A above lane 6–10). Fig 4A above lane 11 is a negative control (H_2_O) and lane 12 a positive control (lung). The bottom lanes of Fig 4A show the respective HPRT1 expression. All SE samples seem to express AQP4 in more or less the same amount. Additionally, we performed Real-Time PCR analysis of AQP4 expression. The Cp-values were normalized to HPRT expression. The results (ΔCp-values) as shown in Fig 5A. The ΔCp-values for the infratentorial growing SEs vary only slightly between– 5.28 and– 6.53. Comparable results were achieved for the supratentorial SEs, which show Comparable results were achieved for the supratentorial SEs, which show ΔCp-values between– 4.7 and– 6.75. Summarized, these results show that the AQP4 expression is upregulated in SEs. In contrast, the Normal Brain Sample showed a ΔCp-values of– 1 (the two values are due to two different PCR-runs), indicating that there is no upregulation of AQP4 compared to HPRT1 expression in the healthy brain. T-Test analysis also revealed no significant difference between infra- and supratentorial samples concerning AQP4 expression (p = 0.8153). Compared to AQP4 expression of the Normal Brain both SE subsets (infra- and supratentorial) showed significant higher gene expression (p<0.0001 and p = 0.0008, respectively). The results showed AQP4 mRNA expression independent of the localization of the tumors.

We therefore conclude that the different distribution patterns revealed by immunohistology are due to different extracellular components of the environment around infra- and supratentorial tumors.

In contrast to AQP4, AQP1 was differentially expressed, with the infratentorial samples showing higher expression than the supratentorial samples. These results could be shown by normal PCR (Fig 4B with the same layout as for AQP4) as well as by RT-PCR (Fig 5B). The infratentorial SE samples showed very distinct bands in the gel, whereas the AQP1 expression in supratentorial SEs varied and where less distinct. These visual results could be confirmed by Real-Time PCR analysis, the infratentorial SE samples showing ΔCp-values between– 1.27 and– 4.49 in the supratentorial SEs having higher ΔCp-values between– 1.44 and 1.83. This indicates that AQP1 is upregulated in infratentorial SEs but not in supratentorial SEs. These results are supported by the results of the Normal Brain, which shows a slight downregulation of AQP1 expression compared to HPRT1 expression. We also performed T-Test analysis for the AQP1 RT-PCR results.

Regarding AQP1 expression both SE subsets showed significantly different results, with p = 0.002. The same is true when comparing infratentorial samples with normal brain samples, the former showing significant higher expression (p = 0.0025). In contrast, there is no significant different AQP1 expression between supratentorial samples and the healthy brain sample detectable (p = 0.1636).

**Fig 6:**
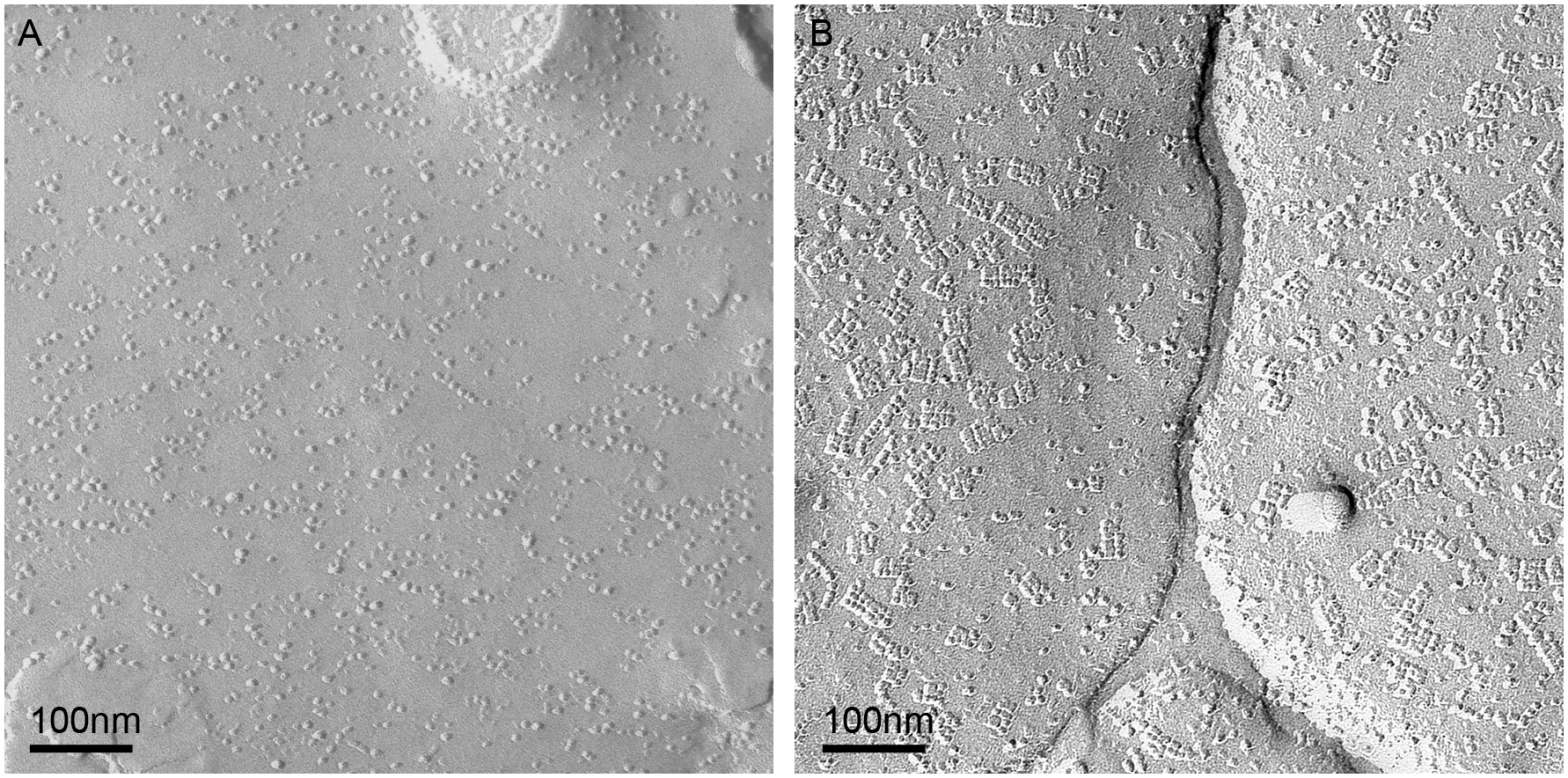
Freeze fracture electron micrograph. A Replica from tissue of patient 5: SE (infratentorial), no OAPs were found, B: Healthy astrocytic endfoot membrane of rat cortex is covered with OAPs. Bar 250 nm.

**Fig 7:**
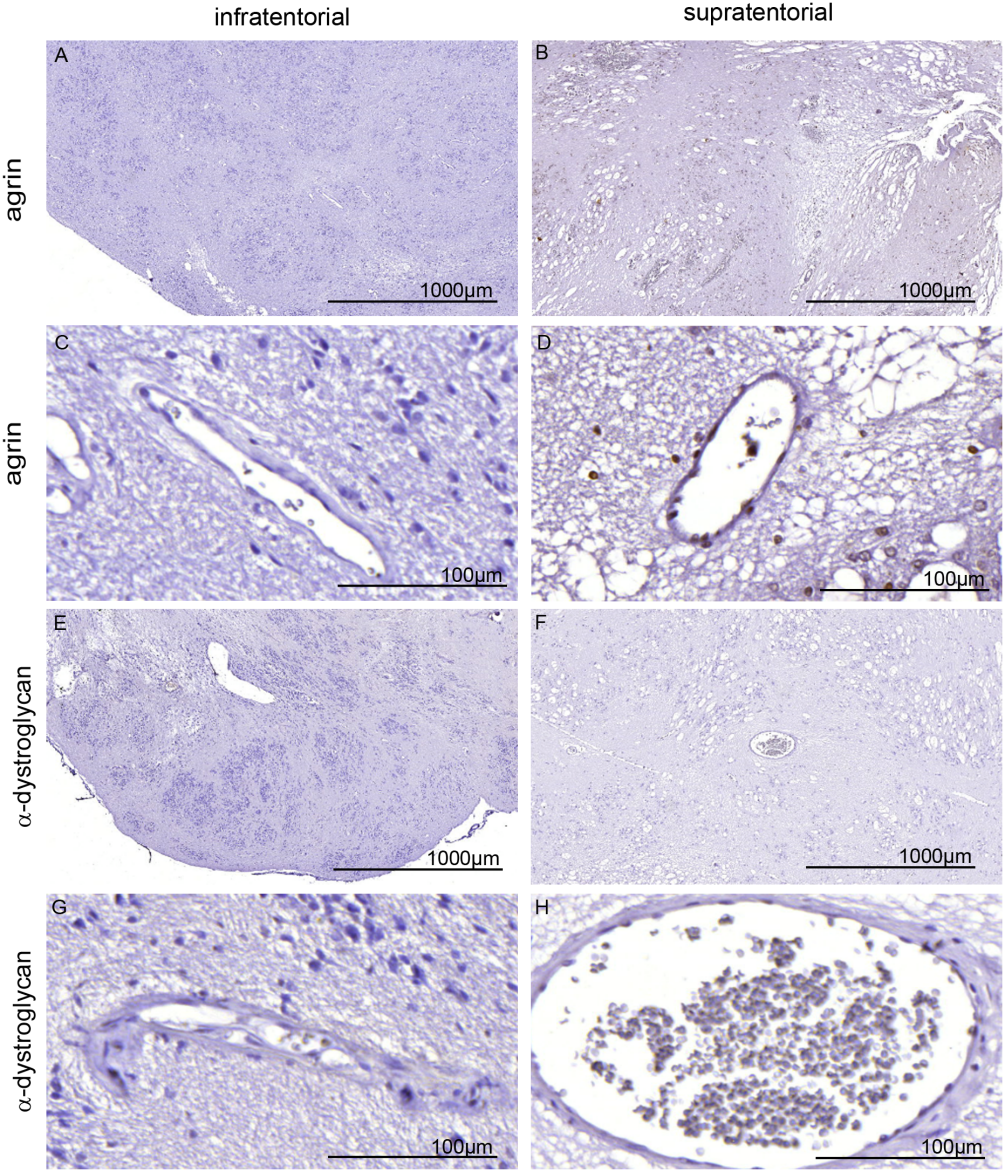
Immunhistological staining of agrin (A-D) and alpha-dystroglycan (E-H) in SE. Neither infratentorial SE (A, C, E, G, patient 5) nor supratentorial SE (B, D, F, G, patient 9) were positive for agrin or dystroglycan, normally found around the blood vessels (for positive control see supplement S3 Fig Bar 1000 and 50μm.

**Fig 8:**
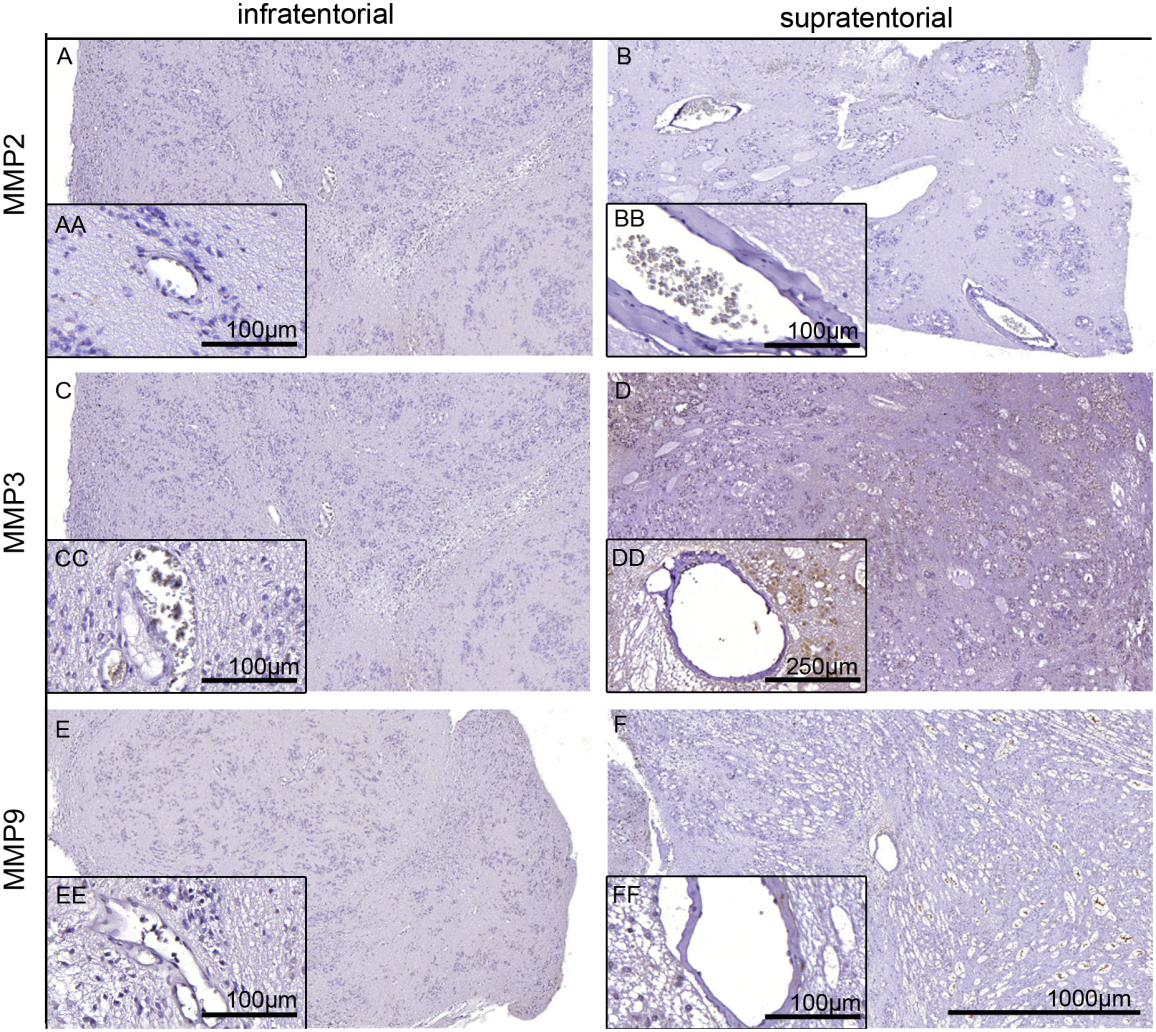
Staining for MMPs in SE tissues. Infratentorial (A, C, E, patient 5) and supratentorial (B, D, F, patient 9) SE tissues were stained with antibodies against MMP2 (A, B), MMP3 (C, D), and MMP9 (E, F). The inserts with double letters show a higher magnification as indicated. None of the immunostains showed significant immunoreactivity. For controls, see S3 Fig.

Next, we used freeze fracture electron microscopy to test for AQP4 forming OAPs and compared samples of all SEs to healthy rat brain tissue (Fig 6B). AQP4 did not form any OAPs in infratentorial SEs (Fig 6A) despite the strong immunoreactivity (c.p. Fig 2). Freeze fracture replica of supratentorial SE also showed no OAPs (data not shown). It is known from our previous studies that the formation of OAPs is dependent on the presence of agrin and α-dystroglycan around the vessels. As shown in Fig 7, neither agrin nor α-dystroglycan could be detected in infratentorial and supratentorial SEs around the blood vessels. MMP2 and 9 as well as MMP3 were also not clearly displayed of any SEs near the vessels in the tumors (Fig 8). Positive and negative controls are depicted in S3 Fig. All results are summarized in Table 4.

**Table 4:** Summary of results.

	GFAP	AQP4	AQP1	Agrin, α-dystroglykan	MMP2and-9,MMP3	OAPs	RT PCR, AQP4	RT-PCR, AQP4
**Infratentorial SE**	+	+	+	-, vessel	-, vessel	-	++	++
**Supratentorial SE**	+	+/- periphery	+/- periphery	- vessel	- vessel	-	++	+

^++^ strong expression

^+/-^ weak expression

^+^ moderate expression

^-^ no expression

This is the first study to show AQP4 and -1 expression in benign SEs, yet with a differential distribution depending on the origin of SE. Infratentorial SEs growing mainly in the region of the fourth ventricle displayed AQP4 and -1 throughout the tumor tissue whereas supratentorial SEs near the lateral ventricles of the cerebrum mostly show AQP4 and -1 at some tissue margins morphologically i in representing the tumor border.

The influence of the environment surrounding the tumors on AQP4 expression was demonstrated by Noell et al. [19], who found AQP4 only in glioma cells implanted into mouse brain but not if implanted into mouse flanks. Assuming that all SEs are of similar origin, the present study suggests that the distribution of AQP4 depends on different environments within the brain. It is still unclear which factors are responsible for the expression as well as for the distribution of AQP4 protein. Analyzing healthy brain tissue is not allowed by the German ethics committee. Alternatively, the original tumor cells might differ between supra- and infratentorial SEs (see below).

Ependymomas are graded higher (WHO grade II and III) and more aggressive than SEs but display comparable growth. Two thirds of the tumors are growing infratentorial, one third supratentorial. Wang and Owler (2011) [25] observed AQP4 expression only in infratentorial ependymomas and not in supratentorial ependymomas. However, in our study, PCR analysis as well as immunohistochemistry of both infra- and supratentorial SEs clearly showed AQP4 expression. The difference between the two locations was found on the histological distribution of AQP4. An alternative explanation is the suggestion by Wang and Owler (2011) [25], that this phenomenon “may reflect a difference in the underlying characteristics of the progenitor cell of the tumor”, supporting the postulate that these tumors of the two localizations belong to two molecularly different subgroups [29]. It is known that astrocytes of the phylogenetic younger brain regions like cerebrum are different from astrocytes of the phylogenetic older brain regions like cerebellum and brain stem [30]. This could also be true for ependymal and choroid plexus cells which could have an influence on the SE in the different regions and consequently on the AQP4 and -1 expression and distribution. Ependymal cells form a continuous cell layer lining the ventricle and covering the blood vessels of the choroid plexus, yet differ in their AQP expression depending on location[24]. Wang and Owler (2011) [25] found less AQP1 expression in supratentorial ependymomas than in infratentorial ependymomas. In this study, besides AQP4, AQP1 was detected in SEs and appeared in infratentorial as well as in supratentorial SEs in the same distribution pattern as AQP4. Within SE tumors the morphological appearance of AQP4 seemed similar to glioblastomas although benign SEs showed less blood-brain barrier impairment than malignant glioblastomas. In our previous studies we could show that AQP4 in glioblastoma cells is redistributed [17,31] over the entire cell surface compared to healthy astrocytic endfoot membranes which display polarized expression of AQP4. Furthermore, astrocytic membranes contacting the glial-endothelial basal lamina in healthy brain tissue show a high density of orthogonal arrays of particles (OAP), the morphological equivalent of AQP4. In glioblastoma AQP4 polarization is lost and typical OAPs are not found. In this study no OAPs could be detected independent of the tumor origin and region. Noell et al. [10,22] demonstrated that both agrin, a heparansulfat proteoglycan of the extracellular matrix, and α-dystroglycan, a member of the DDC-complex in the astrocyte membrane, were necessary for the formation of OAPs in mouse brains. This is consistent with the investigation of glioblastomas [17] where it was shown that matrix-metallo-proteinases MMP3 cleaved agrin, and MMP2 and -9 destroyed α-dystroglycan around the blood vessels. Consequently no typical OAPs could be verified. In the present study, agrin and dystroglycan as well as MMP3, 2 and 9 could not be observed around the vessels of SEs. Therefore, we conclude that the blood vessels of SEs differ in their architecture from normal brain vessels.

## Conclusions

We conclude that there are two different influences on the expression and distribution of AQP4 and -1: firstly the influence of the ECM around the vessels within SEs showing no agrin and dystroglycan expression and therefore no OAPs in the tumor cell membranes. Secondly, the influence of the supratentorial and the infratentorial environment around SEs might cause a different AQP4 and AQP1 distribution pattern in SEs.

In addition we summarize that malignant glioblastoma cells as well as benign SE cells show a redistribution of AQP4 and -1 expression. However, there is no formation of edema in SE in contrast to glioblastoma and also less migration and infiltration of SE cells compared to glioma cells. Consequently, AQP4 redistribution and AQP1 upregulation cannot be used as markers to indicate malignancy in these tumors.

## Supporting Information

S1 Fig AQP4 stain on tissues of all patients investigated and control stains. All infratentorial tumor samples (A, patient 4, C, patient 3, E, patient 2, and G, patient 1) show evenly distributed immunoreactivity throughout the tissue, whereas samples derived from supratentorial tumors (B, patient 8, D, patient 10, F, patient 7, H, patient 9) immunoreactivity was restricted to the border areas of the tumor. I: negative control without primary antibody. J: positive control from a rat brain sample shows clear perivascular and subpial staining. Due to ethical restrictions, human healthy brain tissue was not available.
(TIF)

S2 Fig AQP1 stain on tissues of all patients investigated and control stains. Infratentorial tumor samples (A, patient 5, C, patient 3, E, patient 2 and G, patient 1) show reveal many immunpositive cells distributed throughout the tumor tissue, whereas in samples derived from supratentorial tumors (B, patient 6, D, patient 10, F, patient 7 and H, patient 9) immunoreactivity was restricted to the border areas of the tumor. I: negative control without primary antibody. J: positive control from a mouse brain choroid plexus revealing apical AQP1 staining in plexus epithelial cells.
(TIF)

S3 Fig Immunohistochemical control stains for MMP-2, -3, -9, agrin, and alpha-dystroglycan. Immunoreactivities for MMP2 (A, B), MMP3 (C, D), and MMP9 (E, F) were performed on thymus tissue and were clearly positive, especially surrounding blood vessel. The negative control without primary antibody is shown in G (thymus), and H for a section through a mouse brain. Positive staining controls for agrin is shown on a mouse brain section (I), and for alpha-dystroglycan on sample of human muscle (J). For stains on tumor tissue, see Fig 7 in the main paper.
(TIF)

S4 Fig Immunohistochemical control stains for AQP4 and GFAP. Control brain tissue from one patient suffering from a meningioma that was removed in order to facilitate the access for tumour resection. AQP4 (green) and GFAP (red) are colocalized and at the astrocytic endfeet around blood vessels.
(TIF)
