# Supplementary figures and images for: Water Channels Aquaporin 4 and -1 Expression in Subependymoma Depends on the Localization of the Tumors
Source: PLoS One. 2015 Jun 26;10(6):e0131367. doi: 10.1371/journal.pone.0131367 (PMC4482577; doi:10.1371/journal.pone.0131367)

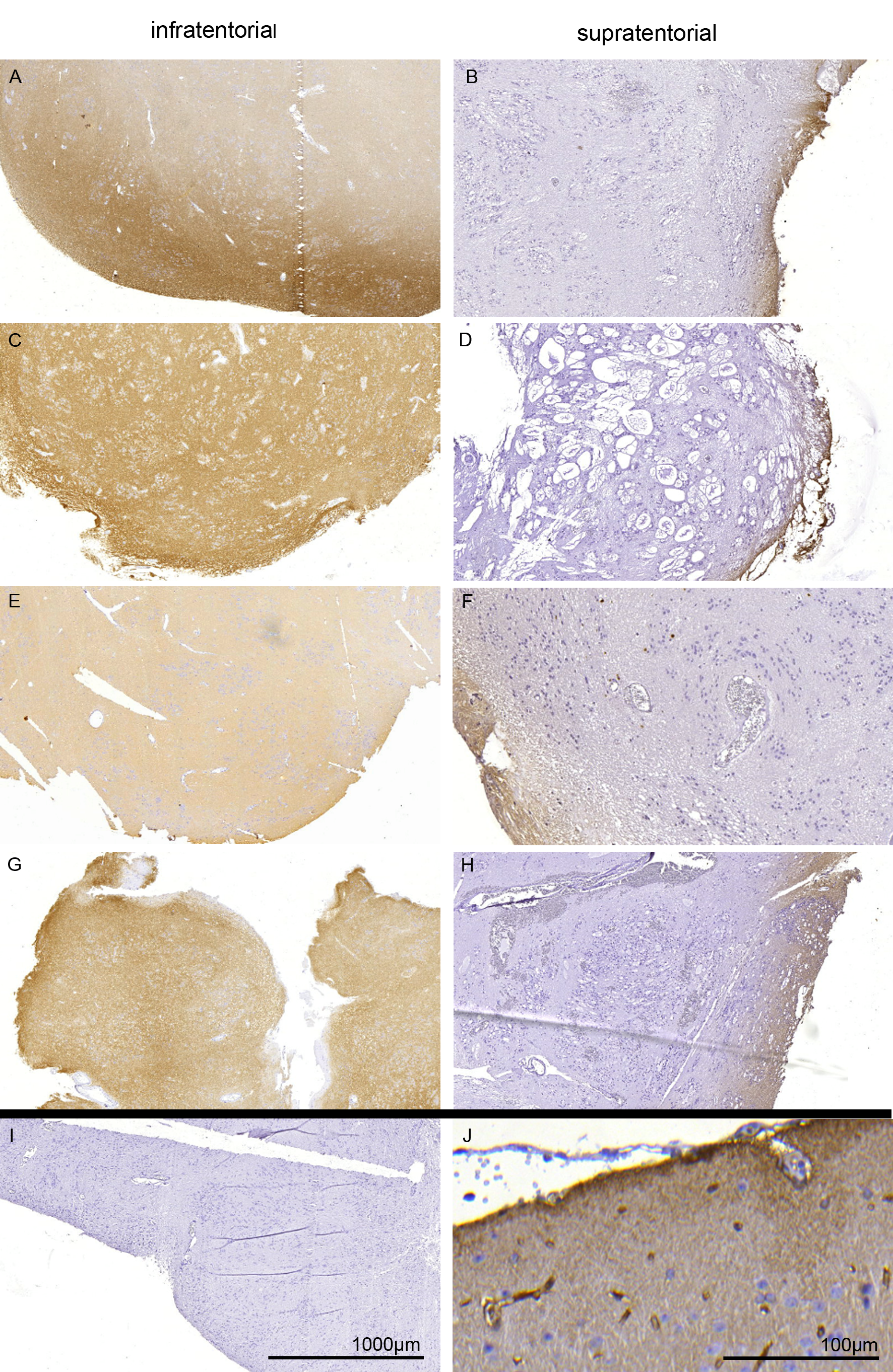

Supplement: S1 Fig — All infratentorial tumor samples (A, patient 4, C, patient 3, E, patient 2, and G, patient 1) show evenly distributed immunoreactivity throughout the tissue, whereas samples derived from supratentorial tumors (B, patient 8, D, patient 10, F, patient 7, H, patient 9) immunoreactivity was restricted to the border areas of the tumor. I: negative control without primary antibody. J: positive control from a rat brain sample shows clear perivascular and subpial staining. Due to ethical restrictions, human healthy brain tissue was not available. (TIF) [file pone.0131367.s001.tif]

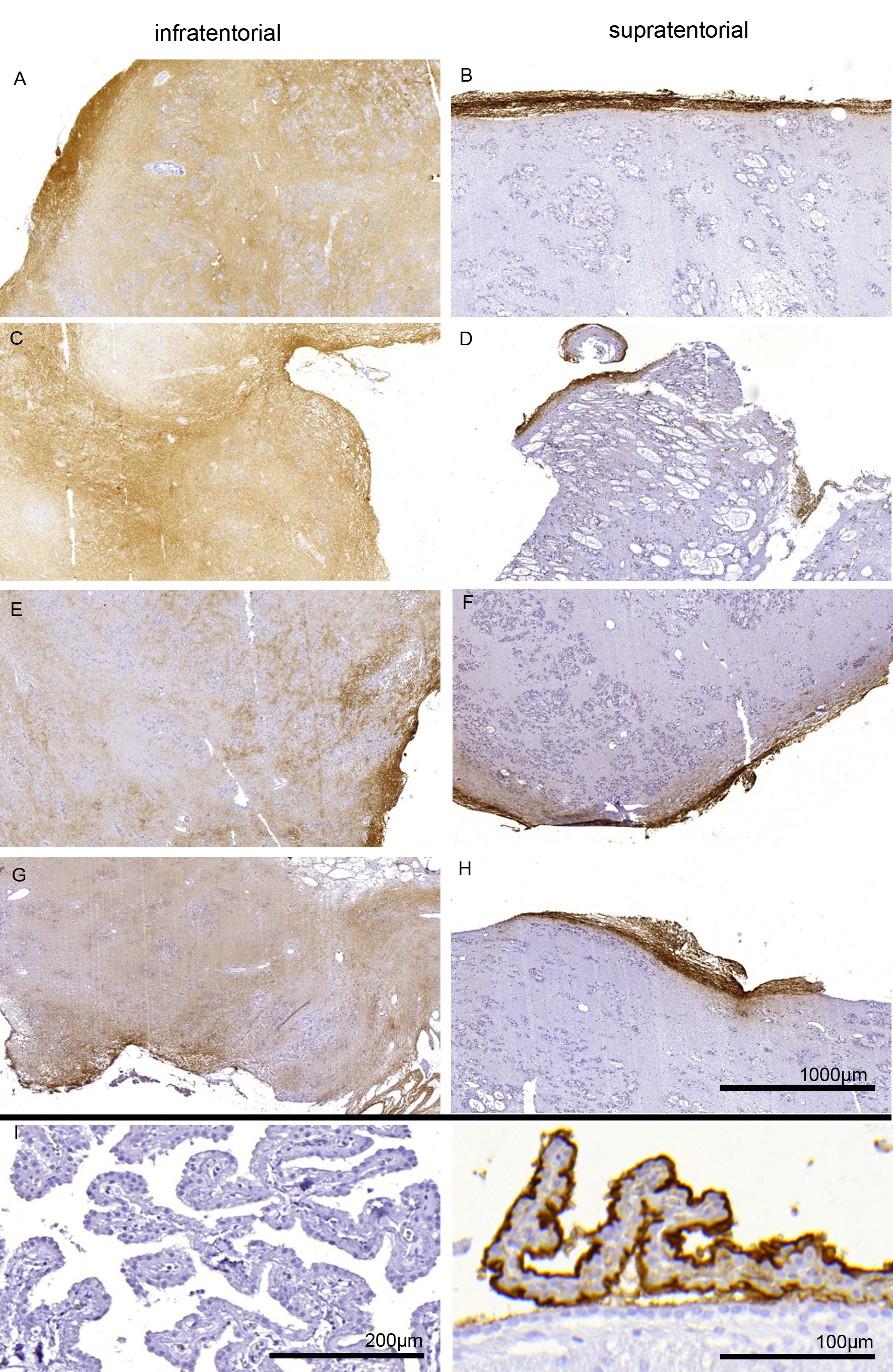

Supplement: S2 Fig — Infratentorial tumor samples (A, patient 5, C, patient 3, E, patient 2 and G, patient 1) show reveal many immunpositive cells distributed throughout the tumor tissue, whereas in samples derived from supratentorial tumors (B, patient 6, D, patient 10, F, patient 7 and H, patient 9) immunoreactivity was restricted to the border areas of the tumor. I: negative control without primary antibody. J: positive control from a mouse brain choroid plexus revealing apical AQP1 staining in plexus epithelial cells. (TIF) [file pone.0131367.s002.tif]

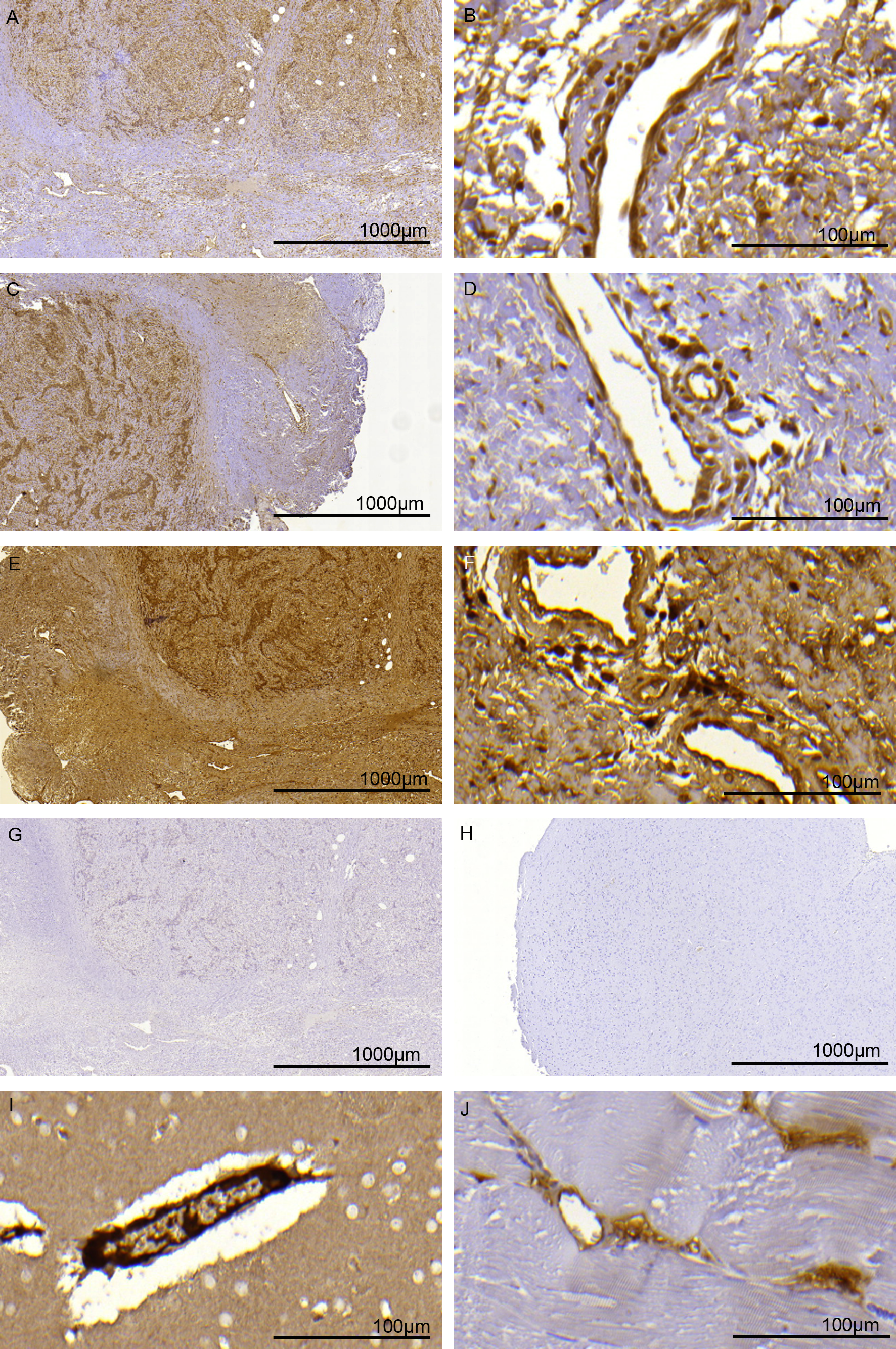

Supplement: S3 Fig — Immunoreactivities for MMP2 (A, B), MMP3 (C, D), and MMP9 (E, F) were performed on thymus tissue and were clearly positive, especially surrounding blood vessel. The negative control without primary antibody is shown in G (thymus), and H for a section through a mouse brain. Positive staining controls for agrin is shown on a mouse brain section (I), and for alpha-dystroglycan on sample of human muscle (J). For stains on tumor tissue, see Fig 7 in the main paper. (TIF) [file pone.0131367.s003.tif]

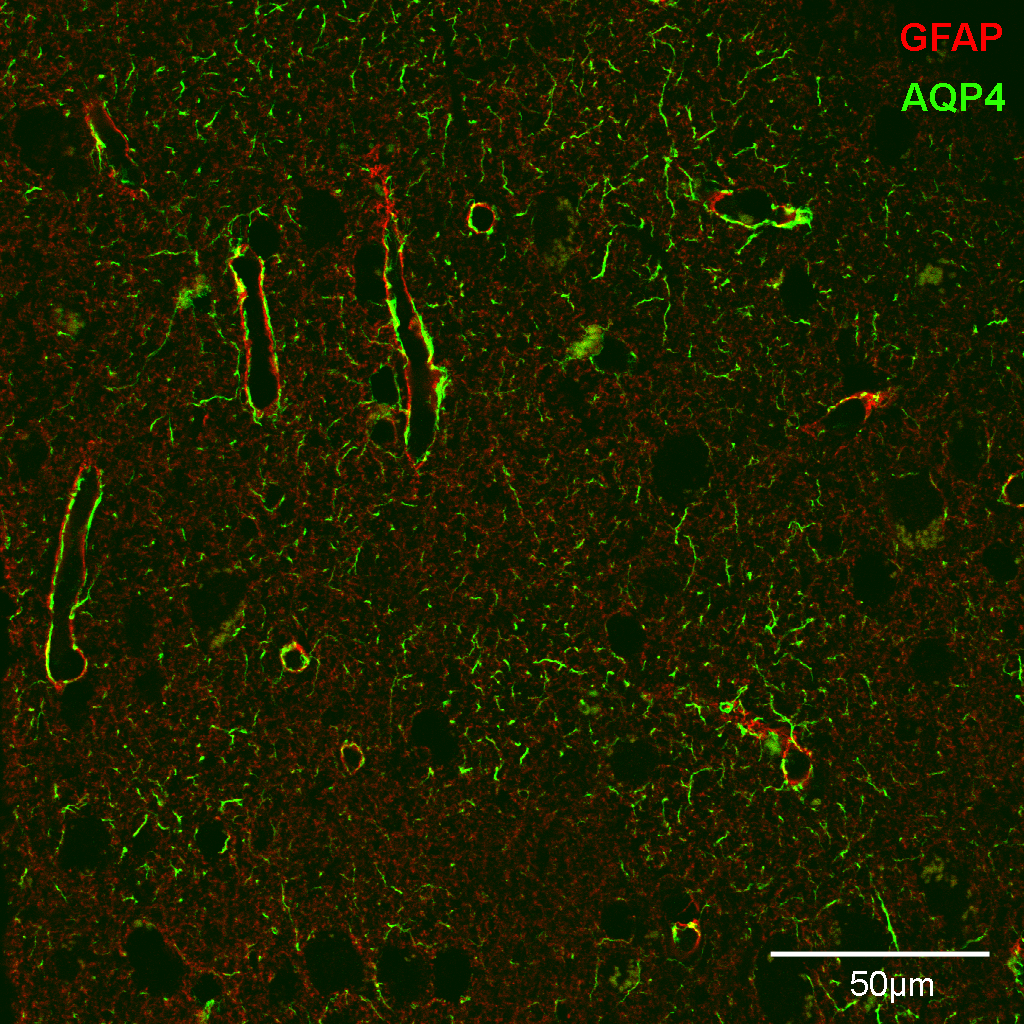

Supplement: S4 Fig — Control brain tissue from one patient suffering from a meningioma that was removed in order to facilitate the access for tumour resection. AQP4 (green) and GFAP (red) are colocalized and at the astrocytic endfeet around blood vessels. (TIF) [file pone.0131367.s004.tif]
